# Two tigers cannot live on the same mountain: The impact of the second largest shareholder on controlling shareholder’s tunneling behavior

**DOI:** 10.1371/journal.pone.0287642

**Published:** 2023-06-28

**Authors:** Jia Liao, Yun Zhan, Xiaoyang Zhao

**Affiliations:** 1 Business School, Huaqiao University, Quanzhou, Fujian, China; 2 School of Economics, Jinan University, Guangzhou, Guangdong, China; Universite de Kairouan, TUNISIA

## Abstract

Under the current corporate governance model, the second largest shareholder (SLS) is a very special, common and important presence, which becomes an important counterweight to the controlling shareholder (CS). Through a game matrix, this paper explains whether the SLS will supervise the CS’s tunneling behavior. Based on this, we empirically examine the effect of the SLS on CS’s tunneling behavior in Chinese listed firms between 2010 and 2020. The results indicate that the SLS significantly inhibits CS’s tunneling behavior. In addition, the heterogeneity analysis reveals that the negative effect of the SLS on CS’s tunneling behavior is concentrated in non-state-owned enterprises (NSOEs) and enterprises located in regions with better business environment. This paper provides a reference for resolving the current "conflict of interest" among multiple large shareholders (MLSs), as well as evidence to support the governance role of the SLS in listed firms with MLSs.

## 1. Introduction

According to principal-agent theory, firms have agency problems of power separation [1] and ownership concentration [2]. Controlling shareholder (CS) may use their position of control to extract private benefits, especially when the control is greater than ownership [3,4]. In Chinese listed firms, ownership is highly concentrated in few large shareholders and dispersed in public [5,6]. CS takes control of the board of directors and even assigns cronies as chairman, thus taking control of the firm resources and major business decisions. In addition, the interests and goals of CS and senior managers overlap to a high degree, so that managers’ business decisions also reflect the will of CS. Therefore, agency problems in Chinese listed firms are usually manifested as divergent and conflicting interests between CS and non-CS [7]. In practice, abuse of control and tunneling by CS on the rights and interests of non-CS occur frequently, and there are many cases of firms being forced to exit due to huge appropriation and tunneling by CS.

**Fig 1:**
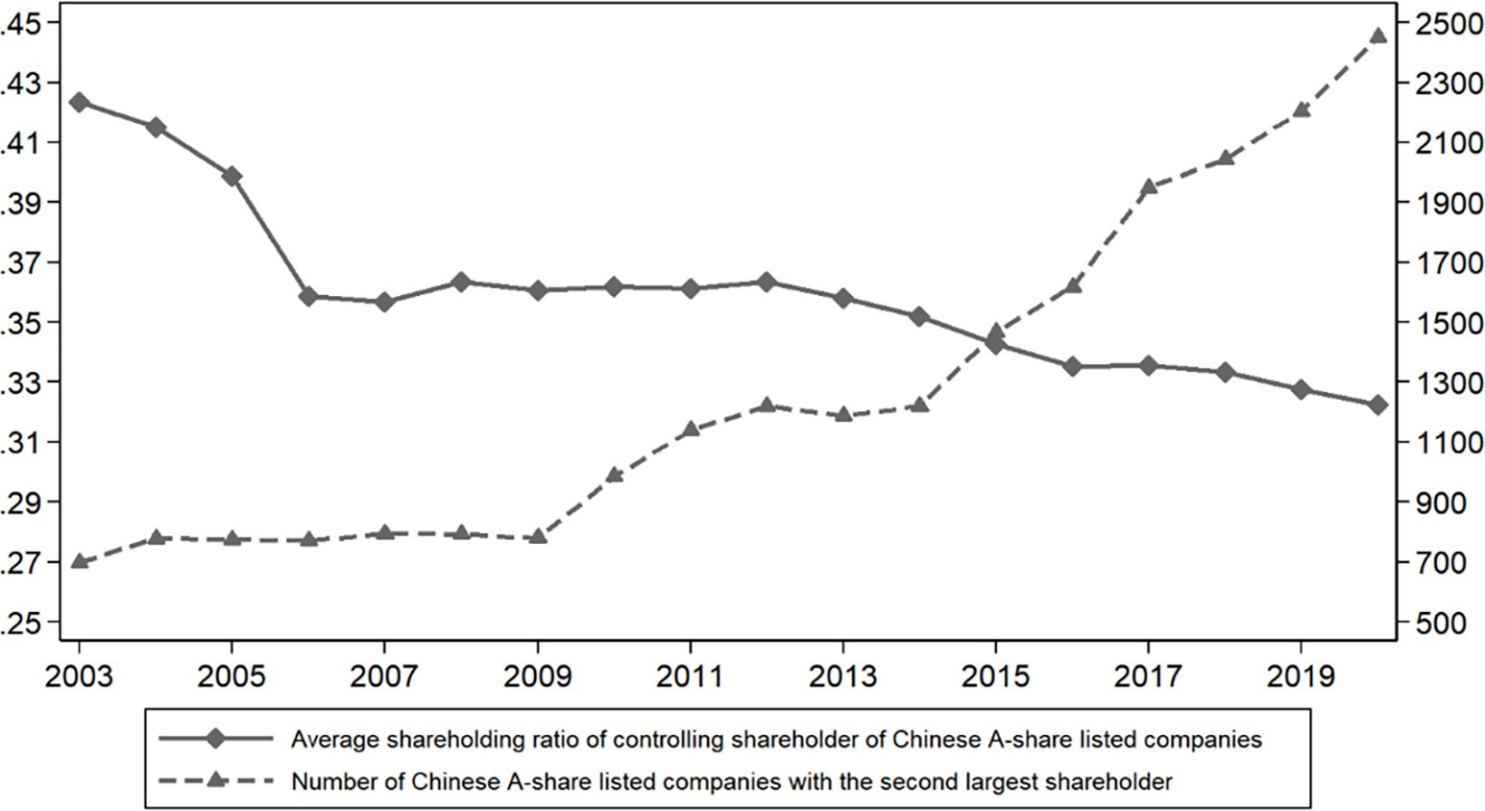
Annual trends in the average shareholding ratio of CS and the number of listed firms with the SLS. The solid line represents the average shareholding of CS and shows a downward trend, and the dashed line represents the number of listed firms with the SLS and shows an upward trend.

During the past ten years, the average shareholding of CS in Chinese listed firms has been decreasing, while the number of listed firms with the SLS has shown a significant upward trend (Fig 1). Under the current corporate governance structure, the SLS is a very special, common and important existence, with the special status of "non-CS" and "non-small and medium shareholders". Bloch and Hege [8] argue that in the optimal ownership structure, the more efficient blockholder will hold just enough shares to gain control, but a large fraction of shares is allocated to the less efficient shareholder in order to reduce rents. Jara-Bertin, López-Iturriaga [9] find that the contestability of control of CS affect the value of the family-owned firms using a sample of firms from 11 European countries. Yan and He [10] use three attributes of the SLS (ownership, identity, and relational board/management representation) to fully capture the governance effects of the SLS, and find that the SLS is associated with higher firm performance. Combined with real cases and literature, the SLS may achieve corporate governance through the "voice mechanism (voting with hands)", the "exit threat mechanism (oral threat)", and the "exit mechanism (voting with feet)". The SLS with higher ownership has the authority to request the convening of extraordinary shareholders’ meetings or directly appoint directors or executives to participate in the operation and management of the firm, and actively participate in corporate governance through "voting with hands" [11,12]. Even if the direct influence through "voting with hands" is ineffective, the SLS can also act as a "bargaining chip" with the controlling shareholder by threatening to sell their ownership stakes [13–15]. Even when both methods fail, the SLS can finally achieve self-rescue by "voting with their feet" [16,17]. At present stage, there is a serious lack of protection mechanism for the small and medium shareholders in Chinese capital market. As the core point of ownership checks and balances, the SLS is an important force that can compete with the CS [18,19]. Whether the SLS can effectively play a governance effect and protect the small and medium shareholders is an important question that needs to be answered. Based on the above background, this paper aims to conduct a detailed and in-depth study on whether and how the SLS affects the CS’s tunneling behavior. The findings provide policy recommendations for listed firms to build a reasonable ownership structure, optimize the corporate governance system, and for regulators to further improve the path of non-controlling large shareholders’ participation in corporate governance.

The contributions and innovations of this paper are: (1) Unlike traditional ownership checks and balances indexes that focus on the "top ten shareholders" [20], this paper focuses on the SLS, who is more likely to actually participate in corporate governance, so the SLS is more in line with the governance practice of listed firms in China. (2) Most previous studies treat the SLS and other large shareholders as a whole, and assume the same values and consistent goals among large shareholders, ignoring the heterogeneity and competition among large shareholders [21]. This paper strictly distinguishes the SLS from CS, internal shareholders and other non-CS, so as to provide more realistic and reliable conclusions on the monitoring and checking effect of the SLS on CS. (3) In view of the possible affiliation or concert party relationships among shareholders [22–24], this paper manually collects information on shareholding relationships, treats concert parties with relationships as the same shareholder, and then combines the shareholding ratios to define the large shareholder, which more accurately portrays the actual shareholding situation of listed firms. (4) Most studies that only measure a single dummy variable of the existence of multiple large shareholders (MLSs) [25,26], this paper portrays the characteristics of the SLS by three indicators: the existence of the SLS, the shareholding ratio of the SLS, and the relative power of the SLS, and the results more accurately reflect the governance role played by the second largest shareholder. (5) Existing studies emphasize the impact of the SLS on firm value or investment decisions [10,27,28], this paper expands the study of the economic consequences of the SLS from the perspective of CS’s tunneling behavior.

The remainder of this paper is organized as follows. Section 2 reviews the relevant literature. Section 3 develops corresponding hypotheses based on the game analysis of the CS and the SLS. Section 4 describes the sample selection process and data sources, variable definitions, and the measurement model. Section 5 presents our baseline results, endogeneity tests, robustness checks, and heterogeneity analysis. Section 6 concludes the paper.

## 2. Literature review

Globally, it is common for listed firms with MLSs structure. La Porta et al. [3] find that 25% of the listed sample firms in 27 countries have more than one large shareholder. Data shows that 34% of European firms [29], 39% of Western European firms [30], 48% of Finnish firms [31] and more than 70% of American firms have MLSs [13,32], and the ownership structure in Malaysia is even directly defined as a MLSs type [33]. In China, since the introduction of the split-share structure reform in 2005, the ownership concentration of state-owned enterprises (SOEs) has been reduced and the ownership structure tends to be balanced and diversified [34].

Numerous studies have shown that ownership structure of MLSs can improve firm performance and enhance firm value. Volpin [25] finds that among Italian listed firms, executive change-performance sensitivity is higher and firm performance is better for firms with MLSs compared to firms with a single large shareholder. Similarly, Maury and Pajuste [31] find that MLSs help to increase firm value, and this effect is more obvious when the shareholding is dispersed and controlled by the family. Jara-Bertin et al. [9], using data on family firms in 11 European countries, find that agency competition induced by ownership structure of MLSs helps to increase firm value. Attig et al. [35] argue that the shareholdings of non-controlling large shareholders in firms with MLSs significantly increase firm value. In addition, there are also studies that verify the positive effect of MLSs on firm performance from various research perspectives. For example, Attig et al. [36] find that MLSs significantly increase firm cash holding value using data from 2,723 firms in 22 countries/regions. Using data from Chinese listed firms, Lin et al. [37] find that the positive effect of MLSs on the value of excess cash holdings is only found in listed firms where the CS is state-owned and the non-controlling larger shareholder is non-state-owned. Based on Chinese listed firms, Ouyang et al. [38] find that the coexistence of MLSs’ ownership structure significantly inhibits firms’ tax avoidance behavior and ultimately enhances firm value by reducing non-tax costs. Besides, Shen et al. [39] take Chinese listed firms as a sample and find that firms with MLSs have a low probability of initiating related-party M&A restructuring transactions, and even they initiate the M&A, the M&A premium paid is lower and the M&A performance is higher, providing evidence to support the positive role of MLSs in listed firms.

In addition to firm financial performance or market value, existing studies have verified the positive effects of MLSs ownership structures on listed firms from other perspectives. Using a sample of Chinese SOEs, Chen et al. [40] find that ownership structure of MLSs helps mitigate agency problems, as evidenced by lower overhead rates and stronger executive compensation-performance sensitivity. Using data from 1686 firms in nine countries, Mishra [26] finds that when a single large shareholder occupies a dominant position, it is more inclined to adopt a conservative strategy to capture private gains in control, resulting in lower levels of corporate risk-taking. While MLSs help to improve the firm’s internal governance, which in turn significantly improves the risk-taking level. Based on Chinese listed firms, Zhao et al. [41] find that MLSs have an negative impact on the probability and frequency of corporate fraud, revealing that non-controlling large shareholders exert an effect in internal governance. Cao et al. [42] examine the role of MLSs in corporate social responsibility and find that the higher the percentage of CS’s shareholding, the poorer the quality of corporate social responsibility reporting, and this rift effect diminishes when there is a greater balance of power between CS and non-CS, especially when CS and non-CS have the same status. Similarly, Wang et al. [43] indicate that MLSs structure significantly contribute to corporate social responsibility fulfillment.

However, some studies indicate that MLSs structure may have negative impacts on firms. Fang et al. [44] find that MLSs are significantly positively associated with management’s excess compensation in Chinese listed firms, revealing the "dark side" of MLSs’ ownership structure, Specifically, coordination frictions among MLSs reduce the efficiency of shareholders’ monitoring of management, which then exacerbates the first type of agency problem. In addition, the presence of MLSs in a firm does not necessarily create a structure of ownership checks and balances, and shareholders may have "collusive motives" to extract private benefits [45,46], and collusion is more likely to be reached among large shareholders with close shareholdings [24,46]. Bennedsen and Wolfenzon [22] point out that large shareholders may form a "community" among themselves through kinship, transaction linkage, and shareholding linkage to take advantage of the interests of minority shareholders. Cheng et al. [23] find that the collusion and tunneling behaviors among MLSs with related relationships are particularly serious for firm value and the interests of small and medium-sized shareholders. Besides, from the perspective of family firms, Lepore *et al*. [47] find that when two families are the CS and the SLS, respectively, collusion behavior arises and firm performance decreases. Similarly, based on the special context of "nepotism" that prevails among the large shareholders of family-owned firms, Sacristán-Navarro et al. [48] indicate MLSs have a greater propensity to collude with family owners and align with the family’s interests.

Under a diversified ownership structure, the SLS of a firm is the most powerful shareholder in addition to the CS. Studying other shareholders and CS as "relationship shareholders" in family firms can explain the failure of ownership checks and balances to a certain extent. However, when there is no "close" relationship between the non-CS and the CS, focusing on the SLS can be an effective entry point for the study of ownership checks and balances. Based on the fact that the second largest shareholder is a key subject that can compete with the CS, some scholars have conducted studies with this focus. Lehmann and Weigand [27] find that the SLS significantly improves firm performance for German listed firms. Using data on Brazilian firms, Crisóstomo *et al*. [49] find that the qualities of corporate governance and board composition are significantly negative related to the CS, while are negative related to with the SLS, which reveals that the SLS can weaken the freedom and power of the CS in the board arrangement. Huyghebaert and Wang [50] indicate that control of the ultimate CS of Chinese firm is positively related to expropriation of minority investor. In contrast, control held by the second to tenth largest shareholders is negatively associated with expropriation of minority investor. In addition, Santos et al. [28] take Western European firms as an example and find that the presence of second and third largest shareholders has a significant positive effect on firm value. Yan and He [10] use the second to third largest shareholders unrelated to the CS as the subject and capture the governance effects of non-controlling large shareholders based on three attributes of these shareholders (sum of shareholdings, status, and board/management representation), and find that non-controlling large shareholders significantly improve firm performance and investment efficiency. From the above analysis, we propose that in Chinese listed firms, due to the relatively concentrated ownership structure, the SLS is the most powerful counterweight to the CS. If the SLS fails to effectively monitor the CS, it will be even more difficult for the remaining shareholders, who are at an information disadvantage and have dispersed voting rights, to play a role. Therefore, this paper argues that the construction of ownership checks and balances represented by the SLS may be effective in promoting corporate governance of listed firms and solving the tunneling problem of the CS.

## 3. The game analysis of the CS and the SLS

The game behavior of the CS and the SLS of the firm is increasingly common and frequent due to the existence of conflict of interest. The CS is often in a dominant position in the game relationship with greater power. However, the SLS will still compete with the CS in order to reduce the loss of possible encroachment of interests, which often puts both sides in a "prisoner’s dilemma". La Porta et al. [51] study the role of the SLS in restraining the self-interest of the CS by establishing a mathematical model, this paper refer to and extend their study and explore the game relationship between the CS and the SLS.

### 3.1 Basic assumptions

- For the sake of analysis, this study first assumes that there exists a CS and a SLS in a listed firm, both holding at least 5% of shares, which is determined in accordance with Chinese securities laws and regulations. The CS and the SLS hold *Y*_*1*_ and *Y*_2_ respectively, of which *Y*_*1*_ > *Y*_2_. The CS has two alternative strategies of "Tunnel" and "Not to tunnel", and the SLS has two alternative strategies of "Confront" and "Not to confront".
- Small and medium-sized shareholders generally have a "free-rider" mentality, and only the SLS is more likely to counter the CS. For the CS, he/she needs to choose certain projects to invest in, and the possible benefit to the CS is *I*_*a*_ and the possible benefit to the SLS is *I*_*b*_.
- Suppose the following costs are considered: first, the CS tends to choose the investment projects that are beneficial to personal interests, and needs to search for information on the distribution of project returns, which requires a certain cost *C*_*1*_. Second, the confrontation of the SLS needs cost *C*_*2*_ to understand the project investments. The third is the penalty cost *C*_*m*_ when the CS is found to have tunnelling behavior. Besides, we assume that *C*_*2*_ < *I*_*b*_, *C*_*1*_ < *I*_*a*_, *C*_*1*_ + *C*_*m*_ > *I*_*a*_.

### 3.2 Scenarios of the game

Based on the above basic assumptions, this study establishes the possible combinations of actions of the CS and the SLS. There are four scenarios and the payoff matrix of both parties is shown in Table 1.

- When the SLS "Confront" and CS "Tunnel", the net profits of the SLS and the CS are *I*_*b*_*—C*_*2*_, *I*_*a*_*—C*_*1*_*—C*_*m*_, respectively.
- When the SLS "Confront" and CS "Not to tunnel", the net profits of the SLS and the CS are -*C*_*2*_, respectively.
- When the SLS "Not to confront" and CS "Tunnel", the net profits of the SLS and the CS are 0, *I*_*a*_*—C*_*1*_, respectively.
- When the SLS "Not to confront" and CS "Not to tunnel", the net profits of the SLS and the CS are 0, 0, respectively.

**Table 1:** Payoff matrix of the CS and the SLS.

		The CS	
		Tunnel	Not to tunnel
The SLS	Confront	(*I*_*b*_*—C*_*2*_, *I*_*a*_*—C*_*1*_*—C*_*m*_)	(- *C*_*2*_, 0)
	Not to confront	(0, *I*_*a*_*—C*_*1*_)	(0, 0)

For the SLS, if he/she knows that the CS will definitely "Tunnel", then since *I*_*b*_*—C*_*2*_ > 0, he/she will "Confront"; if he/she knows that the CS will definitely "Not to tunnel", then since*—C*_*2*_ < 0, he/she will "Not to confront". For the CS, if he/she knows that the SLS will definitely "Confront", then since *I*_*a*_*—C*_*1*_*—C*_*m*_ < 0, he/she will "Not to tunnel"; if he/she knows that the SLS will definitely "Not to confront", then since *I*_*a*_*—C*_*1*_ > 0, the CS will "Tunnel". Thus, the optimal choice of the top two shareholders depends entirely on the behavior and reaction of the other party. In general, when the CS chooses "Tunnel", it is certainly more cost-effective for the SLS to choose "Confront". While the SLS chooses "Confront", the CS will certainly choose "Not to tunnel". This causal loop can never be stopped, no matter from which side it starts. It can be judged that there is no pure strategic Nash equilibrium in this game.

Now we assume that the "Tunnel" probability of the CS is *E*_*1*_, the "Confront" probability of the SLS is *E*_*2*_.

When *E*_*1*_ *is* given, the expected profits of the SLS "Confront" and "Not to confront" are:

$$U_{2}(E_{2},E_{1})=(I_{b}‐C_{2})*E_{1}+(‐C_{2})*(1‐E_{1})$$ (1)

$$U_{2}(1‐E_{2},E_{1})=0$$ (2)

When there is no difference between "Confront" and "Not to confront", we get *U*_*2*_ (*E*_*2*_, *E*_*1*_) = *U*_*2*_ (1*—E*_*2*_, *E*_*1*_). The solution is *E*_1_* = C_2_/*I*_*b*_, which indicates that the key to minimizing the "Tunnel" probability of the CS, *E*_1_*, is to increase the "Confront" benefit and reduce the "Confront" cost for the SLS. In addition, the "Tunnel" probability of the CS depends on the "Confront" cost and benefit of the SLS rather than the "Tunnel" cost and benefit.

When *E*_*2*_ *is* given, the expected profits of the CS "Tunnel" and "Not to tunnel" are:

$$U_{1}(E_{1},E_{2})=(I_{a}‐C_{1}‐C_{m})*E_{2}+(I_{a}‐C_{1})*(1‐E_{2})$$ (3)

$$U_{1}(1‐E_{1},E_{2})=0$$ (4)

When there is no difference between "Tunnel" and "Not to tunnel", we get *U*_*1*_ (*E*_*1*_, *E*_*2*_) = *U*_*1*_ (1*—E*_*1*_, *E*_*2*_), the solution is *E*_2_* = (*I*_*a*_*—C*_*1*_) /*C*_*m*_, which indicates that the key to minimizing the "Confront" probability of the SLS, *E*_2_*, is to reduce the net profit of "Confront" and increase the penalty cost of "Confront". Similarly, it is worth noting that the "Confront" probability of the SLS depends on the "Tunnel" cost and benefit of the CS rather than the "Confront" cost and benefit.

From the results of game, we find that the "Confront" cost and benefit of the SLS, and the "Tunnel" cost and benefit and penalty cost of the CS will directly affect the further decision of the other party. In the process of the SLS’s "Confront" choice, it can be seen that the CS’s behavior orientation will have an important impact on the SLS’s "Confront" choice. When the CS chooses "Tunnel", the SLS is bound to choose "Confront" strategy. And in the process of CS’s "Tunnel" choice, the behavior orientation of the SLS has an important influence on the CS’s "Tunnel" choice. When the SLS chooses "Not to confront", the CS will definitely choose "Tunnel". For example, the CS can choose the project investment according to his own will and take actions against the interests of the firm.

When the CS is able to seek a larger "Tunnel" benefit at a lower cost, or when the SLS is able to obtain a larger "Confront" benefit at a lower cost, the possibility of the SLS’s "Confront" will be significantly increased. However, in the actual process, the cost and benefit of "Tunnel" by the CS is the expected judgment before the "Tunnel", and then the actual cost and benefit in the real "Tunnel" process will affect the expected judgment of the cost and benefit of the next "Tunnel", thus forming a "price illusion" model in the "Tunnel" process. Therefore, the first step to solve the CS’s "Tunnel" is to use this "Tunnel" cost and benefit to form a wrong price model, thus reducing the probability of "Tunnel" and enhancing the benefit of the SLS s. In conclusion, the above game analysis illustrates the need for the SLS to provide supervisory. In other words, when the CS engages in "Tunnel", the SLS must choose a "Confront" strategy. Therefore, we propose:

*H1*: *The SLS decreases the CS’s tunnelling behavior of the listed firm*.

## 4. Research design

### 4.1 Sample selection and data sources

This study collects the data from the China Stock Market & Accounting Research Database (CSMAR). Table 2 presents our data selection process. We initially select A-share firms listed on the Shanghai and Shenzhen Stock Exchanges in China during 2010–2020 as the research sample. In order to ensure the accuracy and stability of the data, we exclude the following firms: (a) firms in financial industry, (b) ST, *ST, PT firms, (c) CS’s shareholdings is less than 5% or CS is not the largest shareholder, and (d) firms with missing values. Finally, we obtain a sample of 27,904 observations.

**Table 2:** Sample selection.

Sample selection process	Observations
A-share firms listed on the Shanghai and Shenzhen Stock Exchanges in China during 2010–2020	32,561
Delete: Firms in the financial industry	(718)
Delete: ST, *ST, PT firms	(1,487)
Delete: Firms with CS’s shareholdings is less than 5% or CS is not the largest shareholder	(411)
Delete: Firms with missing values	(2,041)
Final sample	27,904

### 4.2 Variable definitions

#### 4.2.1 Tunnel of CS (*Tunnel*)

Existing studies mainly use the connected transactions of CS as the main measure of tunnelling behavior. We emphasize the most typical connected transactions, connected purchases and sales, and measure the degree of connected transactions as the ratio of total connected purchase and sale transactions to total assets (*Tunnel*).

#### 4.2.2 The second largest shareholder (*Top2*)

In this paper, the SLS is defined as an external investor whose combined shareholding with its concert parties exceeds 5% and is second only to the CS of the listed firm, and is not the CS or the internal large shareholder (i.e., managers and family members) and its concert parties. In practice, shareholders of some Chinese listed firms may jointly hold shares by entering into a concert party agreement and express their demands by means of taking concerted action when exercising shareholders’ voting rights. Therefore, in order to exclude the inconsistency between shareholders’ shareholdings and voting rights, this paper manually collects information on shareholding relationships, treats concerted actors as the same shareholder, and then combines the shareholdings to define the SLS.

In order to more accurately reflect the governance role played by the SLS, this paper also sets the following three variables to portray the characteristics of the SLS: (a) the existence of the SLS (*Dum*): if the SLS exists in the firm, the value is 1, otherwise it is 0, (b) the shareholding ratio of the SLS (*Ratio*): the combined shareholding ratio of the SLS and its concert parties in the firm, and (c) relative power of the SLS (*Power*): the ratio of the shareholding ratio of the SLS to the shareholding ratio of the CS.

### 4.3 Measurement model

To test H1, the following regression model is constructed in this paper.

$$Tunnel_{i,t}=\alpha_{0}+\alpha_{1}Top2_{i,t}+\alpha_{2}Top1_{i,t}+\alpha_{3}Size_{i,t}+\alpha_{4}Lev_{i,t}+\alpha_{5}Roa_{i,t}+\alpha_{6}Growth_{i,t}+\alpha_{7}Indep_{i,t}+\alpha_{8}BOD_{i,t}+\alpha_{9}BOS_{i,t}+\sum Industry+\sum Year+\varepsilon_{i,t}$$ (5)

where the explanatory variable *Tunnel* is the degree of CS’s tunnelling and the explanatory variable *Top2* is the SLS. Referring to the existing literature [52], this paper controls for financial characteristics and corporate governance variables that may have an impact on the degree of CS’s tunnelling, and controls for industry and year fixed effects, and the variable definitions and measures are detailed in Table 3. The coefficient of *Top2*, *α*_*1*_, in Eq (5), is the main parameter to be estimated, and if H1 holds, then its coefficient estimate should be significantly negative.

**Table 3:** Variables definitions and calculation methods.

Variables	Label	Calculation methods
Dependent variable	*Tunnel*	The ratio of total connected purchase and sale commodity transactions to total assets
Independent variables	*Dum*	If the SLS exists, the value is 1, otherwise it is 0
	*Ratio*	The combined shareholding ratio of the SLS and its concert parties
	*Power*	The shareholding ratio of the SLS to that of the CS
Control variables	*Top1*	The shareholding ratio of the CS
	*Size*	Natural logarithm of total assets
	*Lev*	The ratio of the total liabilities to total assets
	*Roa*	The ratio of net profit to total assets
	*Growth*	Growth rate of the firm’s operational revenue
	*Indep*	The ratio of the number of independent directors to the directors
	*BOD*	Natural logarithm of the number of directors
	*BOS*	Natural logarithm of the number of supervisors

## 5. Empirical results and analysis

### 5.1 Descriptive statistics

The descriptive statistics of the variables reported in Panel A of Table 4 show that the mean value of *Tunnel* is 0.023, the median is 0, the standard deviation is 0.086, and the difference between the maximum and the minimum is 0.722, indicating that there are significant differences in CS’s tunnelling among different firms. The mean values of the *Dum* and the *Ratio* are 0.601 and 0.077, respectively, indicating that the SLS exists in about 60.1% of the listed firms in the sample, while the average value of the sum of the SLS is only 7.7%. Although the SLS generally exists in the sample firms, its shareholding is not high, which reveals that the phenomenon of "domination of a single shareholder" is more prominent in China’s capital market. The mean value of the relative *power* of the SLS is 0.264, the median value is 0.184, and the standard deviation is 0.289, and the difference between the maximum and the minimum is 0.981, indicating that the shareholding ratio of the SLS to the CS varies greatly among firms.

**Table 4:** Descriptive statistics and test for differences in means between groups.

Panel A: Descriptive statistics						
Variable	Observations	Mean	Median	Sd	Min	Max
*Tunnel*	27904	0.023	0	0.086	0	0.722
*Dum*	27904	0.601	1	0.490	0	1
*Ratio*	27904	0.077	0.068	0.080	0	0.317
*Power*	27904	0.264	0.184	0.289	0	0.981
*Top1*	27904	0.372	0.355	0.150	0.103	0.759
*Size*	27904	22.190	22.020	1.282	19.560	25.890
*Lev*	27904	0.430	0.424	0.206	0.053	0.889
*Roa*	27904	0.035	0.036	0.063	-0.278	0.187
*Growth*	27904	0.168	0.105	0.411	-0.626	2.581
*Indep*	27904	0.375	0.357	0.054	0.125	0.571
*BOD*	27904	2.131	2.197	0.200	1.609	2.708
*BOS*	27904	1.235	1.099	0.243	1.099	1.946
Panel B: Test for differences between groups						
	*Top2* = 1		*Top2* = 0		Mean-Diff	
*Tunnel*	0.017		0.031		-0.013***	

Note(s)

*p < 0.1

**p < 0.05

***p < 0.01.

In addition, this paper also groups the firms according to the *Dum*, and the results of the mean difference test between the groups are presented in Panel B of Table 4. The results show that the mean value of *Tunnel* in the group without the SLS is 0.031, while the mean value of *Tunnel* in the group with the SLS is 0.017, which is smaller than the former, and the differences are all significant at the 1% level, indicating that firms with the SLS have less CS’s tunnelling behavior compared to firms without the SLS.

### 5.2 Correlation analysis

The results of correlation analysis among variables reported in Table 5 show that *Dum*, *Ratio*, and *Power* are significantly and negatively correlated with *Tunnel* at the 1% level (Pearson correlation coefficients of -0.076, -0.038, and -0.052, respectively, and Spearman correlation coefficients of -0.083, -0.059, -0.075, respectively). In addition, considering that the correlation coefficients among individual variables are greater than 0.5, this paper uses variance inflation factor (VIF) method and finds that the maximum value of VIF is 1.68 and the average VIF value of all variables is 1.41, showing that there is no serious multicollinearity among variables.

**Table 5:** Correlation matrix.

	*Tunnel*	*Dum*	*Ratio*	*Power*	*Top1*	*Size*	*Lev*	*Roa*	*Growth*	*Indep*	*BOD*	*BOS*
*Tunnel*		-0.083***	-0.059***	-0.075***	0.094***	0.136***	0.137***	-0.039***	0.026***	-0.063***	0.136***	0.166***
*Dum*	-0.076***		0.877***	0.877***	-0.247***	-0.064***	-0.103***	0.080***	0.057***	-0.01	0.015**	-0.077***
*Ratio*	-0.038***	0.787***		0.939***	-0.229***	-0.029***	-0.083***	0.084***	0.049***	-0.004	0.033***	-0.042***
*Power*	-0.052***	0.743***	0.872***		-0.445***	-0.049***	-0.080***	0.043***	0.040***	-0.016***	0.040***	-0.052***
*Top1*	0.088***	-0.261***	-0.204***	-0.478***		0.135***	0.012**	0.146***	0.019***	0.034***	-0.007	0.059***
*Size*	0.072***	-0.042***	0.028***	-0.020***	0.172***		0.515***	-0.071***	0.024***	-0.022***	0.242***	0.272***
*Lev*	0.090***	-0.103***	-0.072***	-0.066***	0.016***	0.509***		-0.422***	0.003	-0.013**	0.141***	0.214***
*Roa*	0.001	0.034***	0.052***	-0.01	0.160***	0.006	-0.356***		0.322***	-0.028***	-0.001	-0.083***
*Growth*	0.009	0.046***	0.035***	0.023***	0.021***	0.032***	0.022***	0.228***		0	-0.002	-0.049***
*Indep*	-0.034***	-0.014**	-0.007	-0.023***	0.048***	0.005	-0.005	-0.033***	-0.002		-0.587***	-0.099***
*BOD*	0.102***	0.013**	0.041***	0.047***	0.003	0.261***	0.145***	0.034***	-0.008	-0.533***		0.305***
*BOS*	0.129***	-0.070***	-0.017***	-0.033***	0.064***	0.288***	0.211***	-0.025***	-0.040***	-0.108***	0.323***	

Lower-triangular cells report Pearson’s correlation coefficients, upper-triangular cells are Spearman’s rank correlation

*** p<0.01

** p<0.05

* p<0.1.

### 5.3 Empirical test of research hypotheses

Table 6 presents the regression results of the SLS’s influence on CS’s tunnelling behavior. The results show that the regression coefficients of *Dum* and *Ratio* are -0.008 and -0.022, respectively, and both are significant at the 1% level, and the regression coefficient of *Power* is -0.003 and significant at the 10% level. These results indicates that: (a) when the SLS exists in a listed firm, the degree of CS’s tunneling is lower, and (b) the greater the percentage of shareholding and relative power of the SLS, the lower the degree of tunneling by the CS. In terms of economic significance, -0.008 denotes that the increase of one standard deviation in *Dum* (0.490) results in a 0.392 percent decrease in *Tunnel* (0.490*0.008), which is equivalent to the decrease of 0.046 standard deviation in *Tunnel* (0.392/(0.086*100)). -0.022 denotes that the increase of one standard deviation in *Ratio* (0.080) results in a 0.176 percent decrease in *Tunnel* (0.080*0.022), which is equivalent to the decrease of 0.020 standard deviation in *Tunnel* (0.176/(0.086*100)). -0.003 denotes that the increase of one standard deviation in *Power* (0.289) results in a 0.087 percent decrease in *Tunnel* (0.289*0.003), which is equivalent to the decrease of 0.010 standard deviation in *Tunnel* (0.087/(0.086*100)). The above results indicate that the SLS can significantly inhibit CS’s tunneling behavior in both statistical and economic terms, which reveals that the SLS can weaken the freedom and self-interest of the CS [49]. Therefore, H1 of this paper is verified.

**Table 6:** Effect of the SLS on CS’s tunneling behavior.

	(1)	(2)	(3)
*Dum*	-0.008***		
	(-7.186)		
*Ratio*		-0.022***	
		(-2.990)	
*Power*			-0.003*
			(-1.774)
*Top1*	0.040***	0.044***	0.044***
	(10.358)	(11.302)	(9.824)
*Size*	0.002***	0.002***	0.002***
	(4.163)	(4.190)	(4.068)
*Lev*	0.031***	0.032***	0.032***
	(9.122)	(9.427)	(9.471)
*Roa*	0.004	0.003	0.002
	(0.428)	(0.338)	(0.255)
*Growth*	-0.001	-0.001	-0.001
	(-0.826)	(-1.066)	(-1.117)
*Indep*	0.017	0.017	0.017
	(1.420)	(1.406)	(1.374)
*BOD*	0.023***	0.023***	0.022***
	(6.465)	(6.327)	(6.284)
*BOS*	0.027***	0.028***	0.028***
	(9.503)	(9.677)	(9.715)
*_cons*	-0.123***	-0.126***	-0.125***
	(-8.040)	(-8.283)	(-8.179)
*Industry*	YES	YES	YES
*Year*	YES	YES	YES
Observations	27904	27904	27904
Adjusted *R*^*2*^	0.077	0.076	0.076

Note(s)

*p < 0.1

**p < 0.05

***p < 0.01 (Robust t- statistics in parentheses).

In addition, the regression coefficients of the control variable *Top1* in columns (1)-(3) are all significantly positive, indicating that Chinese firms have concentrated ownership with the effect that the central agency problem emanates from controlling shareholders expropriating non-controlling shareholders, a phenomenon referred to as ’tunneling’ [53–55]. However, the SLS has the motivation and ability to actively participate in corporate governance [11,12,16–19], effectively inhibit CS’s tunneling behavior.

### 5.4 Robustness test

#### 5.4.1 Firm fixed effects model

In the above analysis, industry and year fixed effects are controlled for in model (1). In order to address the omitted variables that do not vary over time but vary with individual firms, this paper further uses the firm fixed effects model, and the results remain consistent with the previous paper and are presented in Table 7.

**Table 7:** Firm fixed effects model.

	(1)	(2)	(3)
*Dum*	-0.004***		
	(-2.715)		
*Ratio*		-0.034***	
		(-2.816)	
*Power*			-0.007**
			(-2.417)
*Top1*	-0.023**	-0.025**	-0.027**
	(-2.175)	(-2.402)	(-2.389)
*Size*	-0.005***	-0.005***	-0.005***
	(-2.823)	(-2.827)	(-2.842)
*Lev*	0.017**	0.017**	0.017**
	(2.354)	(2.324)	(2.369)
*Roa*	0.019**	0.020**	0.019**
	(2.101)	(2.143)	(2.114)
*Growth*	0.003**	0.003***	0.003***
	(2.574)	(2.655)	(2.582)
*Indep*	0.025*	0.025*	0.026*
	(1.650)	(1.668)	(1.671)
*BOD*	0.014**	0.014**	0.014**
	(2.113)	(2.151)	(2.136)
*BOS*	0.009	0.009	0.009
	(1.374)	(1.378)	(1.346)
*_cons*	0.108**	0.109**	0.110***
	(2.549)	(2.563)	(2.595)
*Industry*	YES	YES	YES
*Year*	YES	YES	YES
*Firm*	YES	YES	YES
Observations	27904	27904	27904
Adjusted *R*^*2*^	0.048	0.048	0.048

Note(s)

*p < 0.1

**p < 0.05

***p < 0.01 (Robust t- statistics in parentheses).

#### 5.4.2 Propensity Score Matching (PSM)

To mitigate the problem of selection bias caused by observable firm characteristics, this paper uses the propensity score matching (PSM) for testing. Based on the presence of the SLS in the firm, this paper divides the sample into treatment group (firms with the SLS) and control group (firms without the SLS), and then conducts 1:1 nearest neighbor matching with all control variables in model (1) as matching variables, and obtains 13,356 valid observations after matching. In Panel A of Table 8, there are significant differences in the characteristics of the treatment and control groups before matching, while the density curves of the two almost overlap after matching. This paper re-runs the regression based on the matched subsamples, and the results remain consistent with the previous analysis and are listed in Panel B of Table 8.

**Table 8:** Propensity score matching method.

Panel A: Density graph before and after matching			
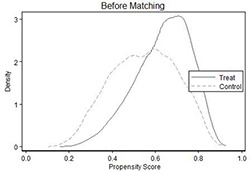		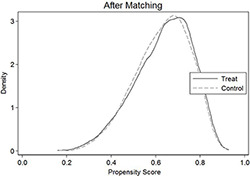	
Panel B: Subsample regression results matching			
	(1)	(2)	(3)
*Dum*	-0.006***		
	(-4.401)		
*Ratio*		-0.026**	
		(-2.443)	
*Power*			-0.006*
			(-1.924)
*Top1*	0.029***	0.028***	0.026***
	(5.440)	(5.299)	(4.646)
*Size*	0.003***	0.003***	0.003***
	(3.232)	(3.433)	(3.388)
*Lev*	0.025***	0.025***	0.025***
	(5.128)	(5.041)	(5.039)
*Roa*	0.004	0.004	0.004
	(0.298)	(0.306)	(0.289)
*Growth*	-0.001	-0.001	-0.001
	(-0.749)	(-0.800)	(-0.796)
*Indep*	0.022	0.022	0.022
	(1.243)	(1.264)	(1.245)
*BOD*	0.028***	0.028***	0.028***
	(5.468)	(5.440)	(5.445)
*BOS*	0.020***	0.020***	0.020***
	(5.314)	(5.362)	(5.323)
*_cons*	-0.128***	-0.132***	-0.130***
	(-5.779)	(-5.970)	(-5.890)
*Industry*	YES	YES	YES
*Year*	YES	YES	YES
Observations	13356	13356	13356
Adjusted *R*^*2*^	0.070	0.069	0.069

Note(s)

*p < 0.1

**p < 0.05

***p < 0.01 (Robust t- statistics in parentheses).

#### 5.4.3 Instrumental variables method

To further address the potential endogeneity between the SLS and the CS’s tunneling behavior, this paper uses the instrumental variables method for regression analysis. Referring to Crisóstomo *et al*. [49], this paper uses the lagged period of the independent variables as the instrumental variables and applies the two-stage least squares (2SLS) regression. The results are shown in Table 9, from which the regression coefficients of the instrumental variables *IVDum*, *IVRatio* and *IVPower* in the first stage are all significantly positive at the 1% level, and F-values are greater than the usual threshold of 10, and the null hypotheses of "under-identification" and "weak instrumental variables" are rejected, which verifies correlation and exogeneity of the above instrumental variable. The second-stage estimation results show that the regression coefficients of *Dum*, *Ratio* and *Power* are significantly negative, indicating that after correcting for the endogeneity bias, the conclusion that the SLS decrease CS’s tunneling behavior still holds.

**Table 9:** Instrumental variable method (2SLS).

	(1)	(2)	(3)	(4)	(5)	(6)
	*Dum*	*Tunnel*	*Ratio*	*Tunnel*	*Power*	*Tunnel*
	First stage	Second stage	First stage	Second stage	First stage	Second stage
*IVDum*	0.800***					
	(224.861)					
*IVRatio*			0.855***			
			(322.947)			
*IVPower*					0.827***	
					(266.568)	
*Dum*		-0.011***				
		(-8.338)				
*Ratio*				-0.034***		
				(-4.641)		
*Power*						-0.007***
						(-2.876)
*Top1*	-0.278***	0.037***	-0.033***	0.043***	-0.246***	0.041***
	(-23.361)	(10.203)	(-22.067)	(11.957)	(-39.807)	(9.786)
*Size*	0.017***	0.003***	0.003***	0.003***	0.010***	0.002***
	(9.951)	(4.830)	(12.228)	(4.848)	(11.712)	(4.707)
*Lev*	-0.083***	0.030***	-0.009***	0.031***	-0.043***	0.032***
	(-7.533)	(9.121)	(-6.176)	(9.488)	(-8.055)	(9.598)
*Roa*	-0.041	0.005	-0.005	0.004	-0.017	0.003
	(-1.323)	(0.521)	(-1.237)	(0.413)	(-1.158)	(0.303)
*Growth*	0.041***	-0.001	0.005***	-0.001	0.014***	-0.001
	(9.758)	(-0.690)	(9.792)	(-1.047)	(6.708)	(-1.107)
*Indep*	0.009	0.018*	0.003	0.018*	0.029*	0.018
	(0.240)	(1.678)	(0.725)	(1.669)	(1.660)	(1.635)
*BOD*	0.007	0.024***	0.001	0.023***	0.010*	0.023***
	(0.663)	(7.269)	(0.419)	(7.069)	(1.926)	(7.043)
*BOS*	-0.009	0.027***	0.001	0.028***	-0.005	0.028***
	(-1.231)	(11.855)	(0.966)	(12.184)	(-1.350)	(12.229)
*_cons*	-0.200***	-0.181***	-0.045***	-0.186***	-0.117***	-0.184***
	(-4.413)	(-13.335)	(-7.798)	(-13.716)	(-5.407)	(-13.573)
*Industry*	YES	YES	YES	YES	YES	YES
*Year*	YES	YES	YES	YES	YES	YES
Observations	27788	27788	27788	27788	27788	27788
Centered *R*^*2*^	0.6824	0.0781	0.8068	0.0771	0.7911	0.0768

Note(s)

*p < 0.1

**p < 0.05

***p < 0.01 (Robust t- statistics in parentheses).

#### 5.4.4 Alternative measures of the CS’s tunnelling behavior

In the previous test, this paper uses connected transactions as a measure of CS’s tunnelling behavior. Considering that existing studies also use CS’s capital appropriation as a measure of tunnelling behavior, this paper use the ratio of other receivables to total assets as a proxy of capital appropriation (*Tunnel2*). The regression results are presented in Table 10. The results show that the regression coefficients of *Dum*, *Ratio*, and *Power* are -0.002, -0.009, and -0.004, respectively, and are significant at the 1% level, indicating that the SLS can significantly inhibit the capital appropriation behavior of CS, and the conclusion of this paper still holds.

**Table 10:** Alternative measures of the CS’s tunnelling behavior.

	(1)	(2)	(3)
*Dum*	-0.002***		
	(-3.953)		
*Ratio*		-0.009***	
		(-4.215)	
*Power*			-0.004***
			(-4.997)
*Top1*	-0.013***	-0.013***	-0.015***
	(-10.684)	(-10.777)	(-10.967)
*Size*	-0.001***	-0.001***	-0.001***
	(-5.351)	(-5.181)	(-5.091)
*Lev*	0.020***	0.020***	0.020***
	(13.177)	(13.136)	(13.054)
*Roa*	-0.059***	-0.059***	-0.059***
	(-11.102)	(-11.097)	(-11.101)
*Growth*	-0.000	-0.000	-0.000
	(-0.512)	(-0.563)	(-0.532)
*Indep*	0.004	0.004	0.004
	(0.925)	(0.971)	(1.024)
*BOD*	-0.001	-0.001	-0.001
	(-0.677)	(-0.681)	(-0.573)
*BOS*	-0.004***	-0.004***	-0.004***
	(-4.970)	(-4.889)	(-4.926)
*_cons*	0.053***	0.052***	0.052***
	(9.884)	(9.706)	(9.786)
*Industry*	YES	YES	YES
*Year*	YES	YES	YES
Observations	27890	27890	27890
Adjusted *R*^*2*^	0.108	0.108	0.108

Note(s)

*p < 0.1

**p < 0.05

***p < 0.01 (Robust t- statistics in parentheses).

#### 5.4.5 Heterogeneity analysis

- (a) Effect of ownership structure: SOEs vs. NSOEs

In the context of China’s unique economic system and institutions, the ownership structure of enterprises has been a topic of concern for Chinese studies [56]. There are significant differences between SOEs and NSOEs in terms of resource allocation methods, ownership structure characteristics, principal-agent model, and corporate governance mechanisms, leading to significant differences in the interest-seeking motives and realization mechanisms of CS. This paper explores the influence of the SLS on the CS’s tunneling behavior from the perspective of ownership structure.

In SOEs, the agency problem is mainly manifested as "owner absence" [57], and the first type of agency problem is more serious, while the second type of agency problem of CS’s tunneling behavior on non-CS is less serious. This is because the CS of SOEs are generally governments, which do not have direct rights to earnings [58] and cannot realize any personal private gains from transferring the wealth of listed firms, and thus have less incentive to use their CS status to tunnel listed firms. In addition, SOEs are subject to stronger supervision and restraint by government departments, and have a higher consciousness of complying with national policies. The CS tend to be more powerful under the influence and control of government departments, and the actual power of the SLS is greatly restrained, thus the SLS has a more limited role in supervising and governing the CS’s tunneling behavior. In contrast, in NSOEs, the CS are usually individuals, families or enterprise groups, etc., which have strong motives to transfer the wealth of listed firms, such as appropriating the resources of listed firms by providing guarantees, conducting non-fair related purchase and sale transactions, and appropriating funds of listed firms without compensation. Therefore, the second type of agency problem is more typical for NSOEs, and the CS’s tunneling behavior on non-CS is heavier. In addition, NSOEs are less influenced by government intervention, and the market-oriented operation mechanism drives the SLS to play a more effective role in inhibiting the CS. Based on the above analysis, this paper concludes that the relationship between the SLS and CS’s tunneling will be affected by the ownership structure, which is reflected that the SLS of SOEs has no effect on CS’s tunneling, while the SLS of NSOEs can significantly inhibit CS’s tunneling.

To test this conjecture, this paper groups the SLS of SOEs and NSOEs by the ownership structure to examine the effect of CS’s tunneling separately. Columns (1)-(3) of Table 11 report the regression results based on the subsample of SOEs, and columns (4)-(6) of Table 11 report the regression results based on the subsample of NSOEs. The results show that in the SOEs group, the regression coefficients of *Dum*, *Ratio*, and *Power* are insignificant, while in the non-SOEs group, the regression coefficients of *Dum*, *Ratio*, and *Power* are significantly negative, indicating that the SLS of non-SOEs has a more important role in inhibiting CS’s tunneling compared to SOEs, supporting the above speculation.

**Table 11:** Effect of ownership structure: SOEs vs. NSOEs.

	(1)	(2)	(3)	(4)	(5)	(6)
	SOEs			Non-SOEs		
*Dum*	-0.003			-0.002***		
	(-1.420)			(-2.697)		
*Ratio*		0.014			-0.013**	
		(0.872)			(-2.575)	
*Power*			0.008			-0.003*
			(1.630)			(-1.876)
*Top1*	0.097***	0.103***	0.108***	0.005*	0.005**	0.004
	(10.140)	(10.687)	(10.051)	(1.818)	(1.992)	(1.346)
*Size*	-0.002*	-0.003**	-0.003**	0.000	0.000	0.000
	(-1.794)	(-2.170)	(-2.238)	(0.411)	(0.448)	(0.482)
*Lev*	0.036***	0.037***	0.037***	0.016***	0.016***	0.016***
	(4.383)	(4.529)	(4.547)	(5.640)	(5.652)	(5.645)
*Roa*	0.042	0.042	0.042	0.013**	0.013**	0.013**
	(1.492)	(1.492)	(1.497)	(1.986)	(2.024)	(1.968)
*Growth*	0.001	0.000	0.000	0.000	0.000	0.000
	(0.214)	(0.146)	(0.123)	(0.414)	(0.378)	(0.354)
*Indep*	-0.015	-0.015	-0.016	0.013	0.013	0.013
	(-0.653)	(-0.668)	(-0.693)	(1.156)	(1.166)	(1.168)
*BOD*	0.036***	0.035***	0.035***	0.001	0.001	0.001
	(5.080)	(4.939)	(4.867)	(0.517)	(0.479)	(0.495)
*BOS*	0.008**	0.008**	0.008**	0.008***	0.008***	0.008***
	(2.026)	(2.003)	(1.995)	(3.152)	(3.174)	(3.191)
*_cons*	-0.036	-0.028	-0.028	-0.005	-0.006	-0.006
	(-1.296)	(-1.024)	(-1.015)	(-0.393)	(-0.451)	(-0.471)
*Industry*	YES	YES	YES	YES	YES	YES
*Year*	YES	YES	YES	YES	YES	YES
Observations	10290	10290	10290	17614	17614	17614
Adjusted *R*^*2*^	0.109	0.109	0.109	0.025	0.025	0.025

Note(s)

*p < 0.1

**p < 0.05

***p < 0.01 (Robust t- statistics in parentheses).

- (b) Effect of business environment: better vs. worse

In recent years, especially since China’s economic development has entered the "new normal", governments at all levels have attached great importance to improving and optimizing the business environment. In the Doing Business 2020 report, China’s Doing Business indicators are most notable for their rapid improvement in the "protection of minority investors" indicator, from 64th in 2019 to 28th in 2020. In China’s capital market, the strongest link to "minority investor protection" is to prevent CS from taking advantage of minority shareholders. However, the business environment in China varies somewhat from province to province. Therefore, the effect of the SLS on the CS’s tunneling behavior should also be considered in the context of the regional business environment.

First, a good business environment implies an equal and clean market environment, which is conducive to enhancing the moral values of market players before engaging in it. The CS’s tunneling is its selfish act of using its absolute voice to hollow out the enterprise in the absence of supervision of the corporate governance system, which is actually an immoral act of appropriating the interests of others to satisfy selfish desires. Therefore, under a good business environment, each economic entity will be influenced by each other, which will drive enterprises and individuals to regulate their own behavior and reduce the probability of CS’s tunneling in advance. Second, a good business environment means a transparent and orderly corporate environment, which is conducive to curbing CS’s tunneling in the process. In a more transparent and orderly corporate business environment, CS’s tunneling behaviors are easily exposed, which objectively puts pressure on CS. At the same time, a transparent information environment is conducive to the SLS to obtain more corporate information and easily identify the CS’s tunneling behavior, so that they can take various ways to actively defend their rights and interests and inhibit the CS’s tunneling. Third, a good business environment means an efficient and perfect rule of law system, which is conducive to increasing the penalty cost of CS after tunneling. Improving the rule of law is an important part of optimizing the business environment, and a good rule of law system can play an effective monitoring role for the core governance issue of CS’s tunneling. This is because a sound rule of law system will prompt external supervisors such as the SLS to react quickly to tunneling, which not only increases the risk of litigation, but also causes great reputational damage to the enterprise and the CS, so an efficient and perfect rule of law system will increase the cost of CS after the tunneling. Based on the above analysis, this paper argues that the negative relationship between the SLS and CS’s tunneling is affected by the business environment, reflecting that in regions with poor business environment, the SLS has insignificant influence on the CS’s tunneling, while in regions with better business environment, the SLS can significantly inhibit the CS’s tunneling.

To test this conjecture, this paper obtains the "government-market relationship" index from the Wind database to measure the level of business environment at the provincial level, and divides the annual median of this index into regions with better business environment and worse business environment, and then conducts a sub-sample test. Since data are only available up to 2016, this paper applies the rankings of the original 2016 index to each year after 2016 on the premise that the cross-province rankings change gradually. Columns (1)-(3) of Table 12 report the regression results based on the better business environment subsample, and columns (4)-(6) of Table 12 report the regression results based on the worse business environment subsample. For firms in regions with better business environment, the SLS has a significantly negative effect on CS’s tunneling behavior, while for firms in regions with worse business environment, the effect of the SLS on CS’s tunneling behavior is not significant, supporting the above speculation.

**Table 12:** Effect of business environment: Better vs. worse.

	(1)	(2)	(3)	(4)	(5)	(6)
	Better			Worse		
*Dum*	-0.009***			-0.002		
	(-7.630)			(-0.662)		
*Ratio*		-0.025***			0.008	
		(-3.087)			(0.503)	
*Power*			-0.004*			0.003
			(-1.865)			(0.637)
*Top1*	0.036***	0.041***	0.040***	0.058***	0.061***	0.063***
	(8.106)	(8.985)	(7.700)	(7.054)	(7.493)	(6.920)
*Size*	0.002***	0.002***	0.002***	0.003**	0.003**	0.003**
	(3.466)	(3.531)	(3.384)	(2.173)	(2.148)	(2.114)
*Lev*	0.032***	0.033***	0.033***	0.023***	0.024***	0.024***
	(8.273)	(8.578)	(8.614)	(3.174)	(3.247)	(3.269)
*Roa*	0.008	0.008	0.007	-0.005	-0.006	-0.006
	(0.823)	(0.754)	(0.681)	(-0.289)	(-0.345)	(-0.327)
*Growth*	-0.001	-0.002	-0.002	0.000	-0.000	-0.000
	(-0.857)	(-1.129)	(-1.177)	(0.021)	(-0.030)	(-0.031)
*Indep*	0.005	0.005	0.004	0.045	0.046	0.045
	(0.393)	(0.367)	(0.308)	(1.586)	(1.608)	(1.602)
*BOD*	0.014***	0.014***	0.014***	0.051***	0.050***	0.050***
	(3.931)	(3.825)	(3.775)	(5.495)	(5.449)	(5.415)
*BOS*	0.031***	0.031***	0.031***	0.011*	0.012*	0.012*
	(9.301)	(9.447)	(9.445)	(1.880)	(1.945)	(1.943)
*_cons*	-0.102***	-0.106***	-0.105***	-0.172***	-0.173***	-0.173***
	(-6.089)	(-6.424)	(-6.276)	(-4.932)	(-4.951)	(-4.939)
*Industry*	YES	YES	YES	YES	YES	YES
*Year*	YES	YES	YES	YES	YES	YES
Observations	22277	22277	22277	5627	5627	5627
Adjusted *R*^*2*^	0.070	0.069	0.068	0.104	0.104	0.104

Note(s)

*p < 0.1

**p < 0.05

***p < 0.01 (Robust t- statistics in parentheses).

## 6. Conclusion and implication

Globally, whether in Western countries with more developed capital market systems or in developing countries that are not yet mature and perfect, the coexistence of MLSs in the shareholding structure arrangement of listed companies is common, and its governance effects and its economic consequences have become a key topic that has attracted extensive attention and lively discussions among scholars in recent years [36–39,59]. However, unlike Western countries, agency problems in Chinese listed companies are more often manifested as divergent interests and conflicting conflicts between CS and non-controlling shareholders. It is a frequent occurrence for CS to abuse its control and take up interests of non-controlling shareholders, and there have been numerous cases of companies being forced to delist due to huge shareholder takeovers and emptying out. Several studies have noted that SLS, as the core point of ownership checks and balances, is an important force that can compete with CS [8,9,18,19]. However, it is still an important question to answer about whether SLS can effectively play the governance effect and restrain the CS’s tunneling behavior in Chinese context.

By building a game model, this paper finds that there are conflicts and disagreements between the CS and the SLS in choosing their interests, and when the CS engages in "Tunnel", the SLS will inevitably choose the strategy of "Confront". Further, this paper empirically examines the influence of the SLS on the CS’s tunnelling behavior by using Chinese A-share listed firms from 2010 to 2020 as the research object. The results show that CS’s tunneling is lower when there is a SLS in the listed firms. In addition, the higher the shareholding ratio and relative power of the SLS, the lower the degree of tunneling by the CS, indicating that the SLS significantly inhibits CS’s tunneling behavior. The above findings hold after overcoming the potential endogeneity problem using fixed effects model, propensity score matching, and instrumental variables approach as well as conducting other robustness tests. In the context of China’s unique economic system and institutions, the ownership structure of enterprises has been a topic of interest in Chinese studies. In this paper, we divide the full sample into SOEs and NSOEs by the ownership structure and find that the SLS of NSOEs has a more significant inhibitory effect on CS’s tunneling compared to SOEs. Moreover, in the current special context of China’s economic transformation, the business environment plays a profound role in influencing corporate behavior. In this regard, this paper divides the annual median of the "government-market relationship" index into regions with better business environment and regions with worse business environment, and finds that the inhibitory effect of the SLS on CS’s tunneling is more significant in regions with better business environment than in regions with poor business environment.

Based on the above findings, this paper puts forward the following policy recommendations: First, listed firms should pay attention to the governance role of the SLS when designing or reforming their ownership structure, especially listed firms controlled by a single large shareholder should actively introduce other large shareholders to further optimize the firm’s ownership structure, and at the same time give the SLS more opportunities to understand the listed firm in depth and actively guide them to participate in corporate governance to maximize the value of the firm. Second, relevant government departments should create a favorable business environment for the SLS to actively participate in corporate governance, such as continuing to actively and steadily promote mixed ownership reform, encouraging the expansion of institutional investors and comprehensively deepening the high-level opening of the capital market to the outside world, so as to attract more high-quality external investors to participate in the governance of listed firms. Meanwhile, the government should increase the liquidity of the stock market and create favorable conditions for outside investors to enter listed firms as influential non-controlling majority shareholders. In addition, the institutional mechanism and regulatory system for investor protection should be improved to effectively protect the legitimate rights and interests of non-controlling large shareholders and increase their willingness to participate in corporate governance. Third, the SLS should actively participate in the governance of the listed firm, strictly review and closely supervise the behavior of the CS and management, and actively express their demands and defend their legitimate rights and interests through the relevant policies and the internal governance mechanism of the listed firm. Besides, the SLS should proactively learn and absorb new professional knowledge and skills, so as to accurately judge whether the business strategy decisions made by the CS and management are scientific and reasonable, and thus improve the level of corporate governance.

## Supporting information

S1 File (ZIP)
