# Supplementary material for: Two tigers cannot live on the same mountain: The impact of the second largest shareholder on controlling shareholder’s tunneling behavior
Source: PLoS One. 2023 Jun 28;18(6):e0287642. doi: 10.1371/journal.pone.0287642 (PMC10306202; doi:10.1371/journal.pone.0287642)
Supplement: S1 File — (ZIP) [file pone.0287642.s001.zip › Supporting Information - CompressedZIP File Archive/Results/Table 10. Alternative measures of the CS's tunnelling behavior.rtf]

variable	N	mean	p50	sd	min	max	
RPT total2 w	28000	0.0230	0	0.0860	0	0.722	
RPT sale2 w	28000	0.0110	0	0.0460	0	0.373	
RPT buy2 w	28000	0.0100	0	0.0440	0	0.382	
Top2dumW w	28000	0.601	1	0.490	0	1	
Top2W w	28000	0.0770	0.0680	0.0800	0	0.317	
Top21W w	28000	0.264	0.184	0.289	0	0.981	
Top1W w	28000	0.372	0.355	0.150	0.103	0.759	
Size w	28000	22.19	22.02	1.282	19.56	25.89	
Lev w	28000	0.430	0.424	0.206	0.0530	0.889	
RoaA w	28000	0.0350	0.0360	0.0630	-0.278	0.187	
Growth w	28000	0.168	0.105	0.411	-0.626	2.581	
ID w	28000	0.375	0.357	0.0540	0.125	0.571	
BS3 w	28000	2.131	2.197	0.200	1.609	2.708	
BOS3 w	28000	1.235	1.099	0.243	1.099	1.946	
