# Supplementary material for: Two tigers cannot live on the same mountain: The impact of the second largest shareholder on controlling shareholder’s tunneling behavior
Source: PLoS One. 2023 Jun 28;18(6):e0287642. doi: 10.1371/journal.pone.0287642 (PMC10306202; doi:10.1371/journal.pone.0287642)
Supplement: S1 File — (ZIP) [file pone.0287642.s001.zip › Supporting Information - CompressedZIP File Archive/Results/Table 5. Correlation analysis.docx]

**相关系数表**

|  | RPT_total2_w | RPT_sale2_w | RPT_buy2_w | Top2dumW_w | Top2W_w | Top21W_w | Top1W_w | Size_w | Lev_w | RoaA_w | Growth_w | ID_w | BS3_w | BOS3_w |
| --- | --- | --- | --- | --- | --- | --- | --- | --- | --- | --- | --- | --- | --- | --- |
| RPT_total2_w |  | 0.889*** | 0.886*** | -0.083*** | -0.059*** | -0.075*** | 0.094*** | 0.136*** | 0.137*** | -0.039*** | 0.026*** | -0.063*** | 0.136*** | 0.166*** |
| RPT_sale2_w | 0.866*** |  | 0.711*** | -0.081*** | -0.056*** | -0.070*** | 0.086*** | 0.127*** | 0.123*** | -0.039*** | 0.018*** | -0.059*** | 0.125*** | 0.151*** |
| RPT_buy2_w | 0.863*** | 0.541*** |  | -0.086*** | -0.056*** | -0.072*** | 0.096*** | 0.164*** | 0.147*** | -0.039*** | 0.021*** | -0.058*** | 0.140*** | 0.182*** |
| Top2dumW_w | -0.076*** | -0.064*** | -0.075*** |  | 0.877*** | 0.877*** | -0.247*** | -0.064*** | -0.103*** | 0.080*** | 0.057*** | -0.010 | 0.015** | -0.077*** |
| Top2W_w | -0.038*** | -0.033*** | -0.035*** | 0.787*** |  | 0.939*** | -0.229*** | -0.029*** | -0.083*** | 0.084*** | 0.049*** | -0.004 | 0.033*** | -0.042*** |
| Top21W_w | -0.052*** | -0.043*** | -0.049*** | 0.743*** | 0.872*** |  | -0.445*** | -0.049*** | -0.080*** | 0.043*** | 0.040*** | -0.016*** | 0.040*** | -0.052*** |
| Top1W_w | 0.088*** | 0.077*** | 0.081*** | -0.261*** | -0.204*** | -0.478*** |  | 0.135*** | 0.012** | 0.146*** | 0.019*** | 0.034*** | -0.007 | 0.059*** |
| Size_w | 0.072*** | 0.053*** | 0.085*** | -0.042*** | 0.028*** | -0.020*** | 0.172*** |  | 0.515*** | -0.071*** | 0.024*** | -0.022*** | 0.242*** | 0.272*** |
| Lev_w | 0.090*** | 0.066*** | 0.100*** | -0.103*** | -0.072*** | -0.066*** | 0.016*** | 0.509*** |  | -0.422*** | 0.003 | -0.013** | 0.141*** | 0.214*** |
| RoaA_w | 0.001 | 0.010* | -0.013** | 0.034*** | 0.052*** | -0.010 | 0.160*** | 0.006 | -0.356*** |  | 0.322*** | -0.028*** | -0.001 | -0.083*** |
| Growth_w | 0.009 | 0.012** | -0.000 | 0.046*** | 0.035*** | 0.023*** | 0.021*** | 0.032*** | 0.022*** | 0.228*** |  | -0.000 | -0.002 | -0.049*** |
| ID_w | -0.034*** | -0.037*** | -0.024*** | -0.014** | -0.007 | -0.023*** | 0.048*** | 0.005 | -0.005 | -0.033*** | -0.002 |  | -0.587*** | -0.099*** |
| BS3_w | 0.102*** | 0.096*** | 0.089*** | 0.013** | 0.041*** | 0.047*** | 0.003 | 0.261*** | 0.145*** | 0.034*** | -0.008 | -0.533*** |  | 0.305*** |
| BOS3_w | 0.129*** | 0.091*** | 0.145*** | -0.070*** | -0.017*** | -0.033*** | 0.064*** | 0.288*** | 0.211*** | -0.025*** | -0.040*** | -0.108*** | 0.323*** |  |
| Lower-triangular cells report Pearson's correlation coefficients, upper-triangular cells are Spearman’s rank correlation | | | | | | | | | | | | | | |
| *** p<0.01, ** p<0.05, * p<0.1 | | | | | | | | | | | | | | |
