# Supplementary material for: Two tigers cannot live on the same mountain: The impact of the second largest shareholder on controlling shareholder’s tunneling behavior
Source: PLoS One. 2023 Jun 28;18(6):e0287642. doi: 10.1371/journal.pone.0287642 (PMC10306202; doi:10.1371/journal.pone.0287642)
Supplement: S1 File — (ZIP) [file pone.0287642.s001.zip › Supporting Information - CompressedZIP File Archive/Results/Table 9. first stage1.rtf]

	(1)	
	Top2dumW_w	
Top1W_w	-0.278***	
	(-23.361)	
		
Size_w	0.017***	
	(9.951)	
		
Lev_w	-0.083***	
	(-7.533)	
		
RoaA_w	-0.041	
	(-1.323)	
		
Growth_w	0.041***	
	(9.758)	
		
ID_w	0.009	
	(0.240)	
		
BS3_w	0.007	
	(0.663)	
		
BOS3_w	-0.009	
	(-1.231)	
		
YEAR1	-0.028***	
	(-3.190)	
		
YEAR2	-0.007	
	(-0.901)	
		
YEAR3	-0.005	
	(-0.668)	
		
YEAR4	-0.007	
	(-0.879)	
		
YEAR5	-0.014*	
	(-1.844)	
		
YEAR6	0.023***	
	(3.114)	
		
YEAR7	-0.001	
	(-0.121)	
		
YEAR8	0.011	
	(1.489)	
		
YEAR9	0.019***	
	(2.829)	
		
YEAR10	0.018***	
	(2.679)	
		
IND1	0.031	
	(1.402)	
		
IND2	0.044**	
	(2.157)	
		
IND3	0.053***	
	(2.838)	
		
IND4	0.052***	
	(2.921)	
		
IND5	0.053***	
	(2.977)	
		
IND6	0.070***	
	(3.293)	
		
IND7	0.079***	
	(3.970)	
		
IND8	0.056***	
	(2.765)	
		
IND9	0.050***	
	(2.666)	
		
IND10	0.061***	
	(3.089)	
		
IND11	0.086***	
	(2.590)	
		
IND12	0.027	
	(1.438)	
		
IND13	0.047**	
	(2.444)	
		
IND14	0.055**	
	(2.395)	
		
IND15	0.055**	
	(2.179)	
		
IND16	0.053**	
	(2.262)	
		
IND17	0.145**	
	(2.199)	
		
IND18	0.069	
	(1.195)	
		
IND19	0.022	
	(0.566)	
		
IND20	0.073***	
	(3.220)	
		
Top2dumW_1	0.800***	
	(224.861)	
		
_cons	-0.200***	
	(-4.413)	
N	27788	
r2	0.682	
r2_a	0.682	
F	1528.698	
t statistics in parentheses
* p < 0.1, ** p < 0.05, *** p < 0.01
