# Supplementary material for: Two tigers cannot live on the same mountain: The impact of the second largest shareholder on controlling shareholder’s tunneling behavior
Source: PLoS One. 2023 Jun 28;18(6):e0287642. doi: 10.1371/journal.pone.0287642 (PMC10306202; doi:10.1371/journal.pone.0287642)
Supplement: S1 File — (ZIP) [file pone.0287642.s001.zip › Supporting Information - CompressedZIP File Archive/Results/Table 9. first stage2.rtf]

	(1)	
	Top2W_w	
Top1W_w	-0.033***	
	(-22.067)	
		
Size_w	0.003***	
	(12.228)	
		
Lev_w	-0.009***	
	(-6.176)	
		
RoaA_w	-0.005	
	(-1.237)	
		
Growth_w	0.005***	
	(9.792)	
		
ID_w	0.003	
	(0.725)	
		
BS3_w	0.001	
	(0.419)	
		
BOS3_w	0.001	
	(0.966)	
		
YEAR1	-0.002*	
	(-1.803)	
		
YEAR2	0.000	
	(0.283)	
		
YEAR3	0.001	
	(1.349)	
		
YEAR4	0.000	
	(0.220)	
		
YEAR5	-0.001	
	(-0.875)	
		
YEAR6	0.004***	
	(3.832)	
		
YEAR7	0.001	
	(1.117)	
		
YEAR8	0.001	
	(1.579)	
		
YEAR9	0.003***	
	(3.594)	
		
YEAR10	0.003***	
	(3.808)	
		
IND1	0.003	
	(0.984)	
		
IND2	0.004	
	(1.515)	
		
IND3	0.004*	
	(1.823)	
		
IND4	0.004*	
	(1.805)	
		
IND5	0.004*	
	(1.684)	
		
IND6	0.005*	
	(1.670)	
		
IND7	0.009***	
	(3.555)	
		
IND8	0.005**	
	(2.043)	
		
IND9	0.005**	
	(2.067)	
		
IND10	0.008***	
	(3.009)	
		
IND11	0.012***	
	(2.846)	
		
IND12	-0.003	
	(-1.126)	
		
IND13	0.004*	
	(1.755)	
		
IND14	0.003	
	(0.926)	
		
IND15	0.005	
	(1.482)	
		
IND16	0.002	
	(0.788)	
		
IND17	0.008	
	(1.014)	
		
IND18	0.012*	
	(1.686)	
		
IND19	-0.000	
	(-0.012)	
		
IND20	0.006**	
	(2.063)	
		
Top2W_1	0.855***	
	(322.947)	
		
_cons	-0.045***	
	(-7.798)	
N	27788	
r2	0.807	
r2_a	0.806	
F	2970.502	
t statistics in parentheses
* p < 0.1, ** p < 0.05, *** p < 0.01
