# Supplementary material for: Two tigers cannot live on the same mountain: The impact of the second largest shareholder on controlling shareholder’s tunneling behavior
Source: PLoS One. 2023 Jun 28;18(6):e0287642. doi: 10.1371/journal.pone.0287642 (PMC10306202; doi:10.1371/journal.pone.0287642)
Supplement: S1 File — (ZIP) [file pone.0287642.s001.zip › Supporting Information - CompressedZIP File Archive/Results/Table 9. first stage3.rtf]

	(1)	
	Top21W_w	
Top1W_w	-0.246***	
	(-39.807)	
		
Size_w	0.010***	
	(11.712)	
		
Lev_w	-0.043***	
	(-8.055)	
		
RoaA_w	-0.017	
	(-1.158)	
		
Growth_w	0.014***	
	(6.708)	
		
ID_w	0.029*	
	(1.660)	
		
BS3_w	0.010*	
	(1.926)	
		
BOS3_w	-0.005	
	(-1.350)	
		
YEAR1	-0.006	
	(-1.358)	
		
YEAR2	-0.001	
	(-0.280)	
		
YEAR3	-0.001	
	(-0.179)	
		
YEAR4	0.000	
	(0.115)	
		
YEAR5	0.000	
	(0.085)	
		
YEAR6	0.012***	
	(3.504)	
		
YEAR7	0.004	
	(1.253)	
		
YEAR8	0.003	
	(0.952)	
		
YEAR9	0.006*	
	(1.806)	
		
YEAR10	0.011***	
	(3.405)	
		
IND1	0.020*	
	(1.849)	
		
IND2	0.027***	
	(2.796)	
		
IND3	0.023**	
	(2.539)	
		
IND4	0.020**	
	(2.385)	
		
IND5	0.025***	
	(2.940)	
		
IND6	0.026***	
	(2.589)	
		
IND7	0.041***	
	(4.280)	
		
IND8	0.028***	
	(2.868)	
		
IND9	0.021**	
	(2.352)	
		
IND10	0.037***	
	(3.851)	
		
IND11	0.039**	
	(2.458)	
		
IND12	0.003	
	(0.282)	
		
IND13	0.023**	
	(2.453)	
		
IND14	0.024**	
	(2.237)	
		
IND15	0.032***	
	(2.686)	
		
IND16	0.017	
	(1.532)	
		
IND17	0.061*	
	(1.938)	
		
IND18	0.047*	
	(1.676)	
		
IND19	-0.004	
	(-0.237)	
		
IND20	0.032***	
	(2.954)	
		
Top21W_1	0.827***	
	(266.568)	
		
_cons	-0.117***	
	(-5.407)	
N	27788	
r2	0.791	
r2_a	0.791	
F	2694.757	
t statistics in parentheses
* p < 0.1, ** p < 0.05, *** p < 0.01
